# Evaluating the Output Performance of the Semiconductor Bridge Through Principal Component Analysis

**DOI:** 10.3390/nano15090672

**Published:** 2025-04-28

**Authors:** Limei Zhang, Yongqi Da, Wei Zhang, Fuwei Li, Jianbing Xu, Li Jing, Qun Liu, Yinghua Ye, Ruiqi Shen

**Affiliations:** 1 Beijing Institute of Space Mechanics & Electricity, Beijing 100094, China; forzhanglimei@sina.com (L.Z.); zw_smilesunshine@foxmail.com (W.Z.); jingli0307@163.com (L.J.); lq0921@hotmail.com (Q.L.); rqshen@mail.njust.edu.cn (R.S.); 2 Department of Applied Chemistry, School of Chemistry and Chemical Engineering, Nanjing University of Science and Technology, Nanjing 210094, China; yongqida0127@163.com (Y.D.); 18251956262@163.com (F.L.); 3 Micro-Nano Energetic Devices Key Laboratory of MIIT, Nanjing 210094, China; 4 Institute of Space Propulsion, Nanjing University of Science and Technology, Nanjing 210094, China

**Keywords:** semiconductor bridge, electric explosion parameter, output capacity, principal component analysis

## Abstract

The complex burst characteristic parameters of SCB were subjected to dimensionality reduction using principal component analysis (PCA), enabling accurate evaluation of the output performance of SCB. The accuracy and reliability of the PCA method were also validated. A 100 μF tantalum capacitor was utilized to excite the SCB, while a digital oscilloscope recorded the characteristic parameters of the SCB explosion. The experimental results demonstrate that the critical burst time of SCB decreases with the rising voltage, and the critical burst energy decreases first and then increases with the rising voltage. The total burst time and total burst energy of SCB all decrease first and then increase with the rise of voltage. The PCA results indicate that as the voltage increases, the score of SCB output capacity initially decreases and then increases, reaching its lowest point at 17 V. The SCB was utilized to ignite lead styphnate (LTNR) under varying circuit conditions; the characteristic parameters obtained were analyzed using PCA to derive comprehensive scores. The same dataset was then input into the PCA model for pure SCB to calculate corresponding comprehensive scores. The consistency between the two sets of scores validated the accuracy and reliability of PCA in assessing SCB output capability.

## 1. Introduction

As the initiating element and energy transfer system of weapon systems, initiating explosive devices have been extensively utilized in various applications such as weapon launching, ammunition propulsion, and attitude control of micro-nano satellites. Semiconductor bridges (SCBs) [1,2,3,4], representing a novel category of pyrotechnic devices, have garnered significant attention in both military and civilian sectors due to their distinctive operating principles and broad application potential [5]. Compared with traditional bridge wire pyrotechnics, SCB pyrotechnics offer enhanced safety and reliability, compact size, rapid response, and superior control over energy output [6,7].

In the explosion process of SCB, the initial event in the bridge area involves the conversion of electrical energy into thermal energy [8,9,10]. As the temperature increases, the bridge material vaporizes and transitions into a plasma state, which enables the SCB to ignite the charge through high-temperature plasma [11,12,13]. Consequently, two coupled energy conversion modes are involved in the ignition process. These modes operate synergistically on the ignition agent, complicating the evaluation of SCB’s output capacity. The SCB explosion process encompasses multiple characteristic parameters, such as critical burst time, critical burst energy, total burst duration, and total burst energy [14,15,16]. These parameters exhibit interdependencies, which add layers of complexity to the analysis. Analyzing a single parameter in isolation often leads to fragmented insights and underutilization of available data. Reducing the number of indices without careful consideration can result in a significant loss of valuable information and potentially erroneous conclusions.

In various research and application domains, it is often necessary to observe data sets containing multiple variables [17,18], collect extensive amounts of data, and analyze them to identify patterns. Multivariate big data sets provide rich information for research and applications but also significantly increase the workload associated with data collection [19,20,21]. Therefore, it is crucial to develop a method that reduces the number of indicators to be analyzed while minimizing the loss of information contained in the original indicators, thereby facilitating comprehensive analysis of the collected data. Given the inherent correlations among variables, transforming closely related variables into fewer new variables can be beneficial. These new variables should be uncorrelated, allowing fewer comprehensive indicators to represent the diverse types of information contained in each variable. Principal component analysis (PCA) is one such dimensionality reduction algorithm [22,23,24,25].

PCA aims to explain most of the variance in the original data using fewer variables. It transforms many highly correlated variables into mutually independent or uncorrelated variables. Typically, a few new variables, known as principal components, which are fewer than the original variables and can explain most of the variation in the data, are selected to serve as comprehensive indicators for interpreting the data [26,27,28].

For the complex explosion process of SCB, PCA can effectively retain most of the original information from the SCB explosion process, thereby enabling a more precise evaluation of the output capacity of SCB. In this paper, a 100 µF tantalum capacitor is utilized to excite the SCB with a voltage gradient ranging from 10 V to 34 V. A digital oscilloscope captures the characteristic parameters of the SCB during the burst process, and these parameters are subsequently summarized and analyzed. To comprehensively evaluate the output capacity of the SCB, PCA is employed to analyze the collected characteristic parameters. Finally, the SCB is used to ignite lead styphnate, thereby validating the accuracy and reliability of PCA in assessing the SCB’s output capacity.

## 2. Experiment

### 2.1. Materials

**Figure 1:**
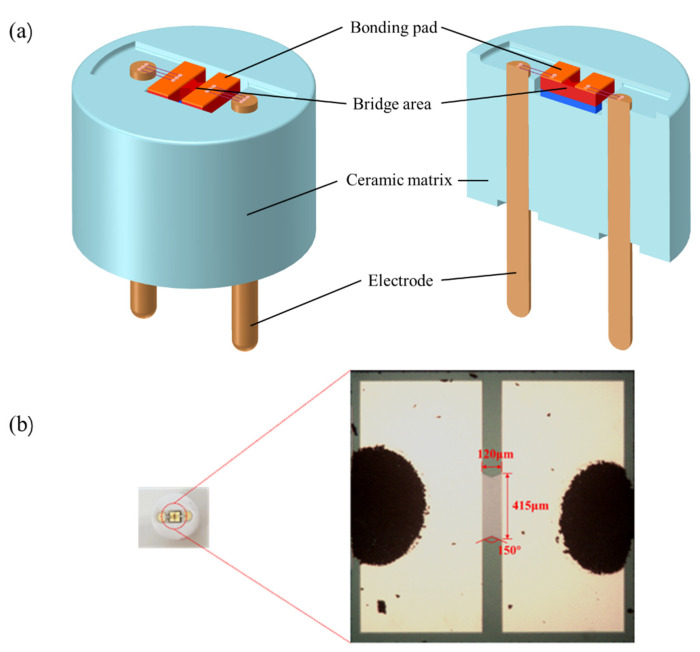
Structure diagram of SCB: (**a**) the schematic diagram of SCB; (**b**) the physical diagram of SCB and dimensional specifications of the bridge region.

The structure of the SCB features a “double V” shape. Figure 1a illustrates the schematic diagram of the SCB, which is encapsulated with a ceramic plug. The pad material of the SCB is Ti/Au, while the material in the double V bridge region is phosphorus-doped polysilicon with a doping concentration of 7 × 10^19^ cm^−3^. Figure 1b shows the physical appearance of the SCB. The central bridge region exhibits a V-shaped angle of 150°, with dimensions of 120 μm × 415 μm × 2.5 μm, and the resistance of the SCB is 1 Ω.

### 2.2. The Firing Test System

**Figure 2:**
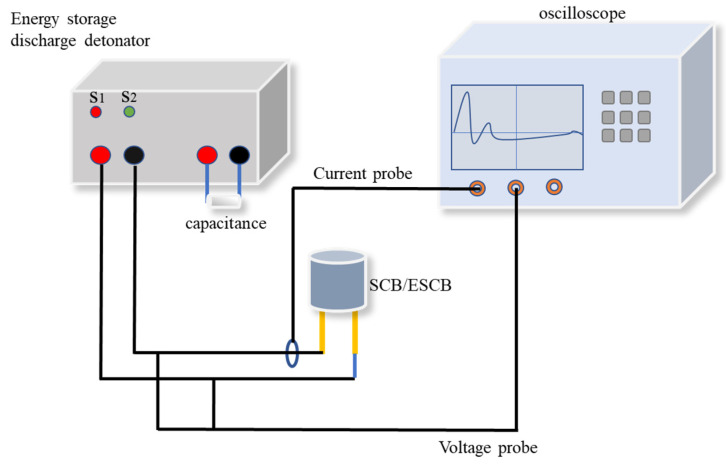
Schematic representation of the SCB electrical initiation device.

The schematic diagram of the experimental setup for detonating SCB using capacitor discharge is illustrated in Figure 2. A tantalum capacitor with a capacitance of 100 μF was employed. The experiment was conducted at voltages ranging from 10 V to 34 V in increments of 1 V up to 24 V, followed by 26 V, 28 V, 30 V, and 34 V, with two parallel experiments performed at each voltage level. During the experiment, the SCB was connected to the circuit. Switch S2 was closed to charge the capacitor to the desired test voltage, after which switch S1 was closed to initiate the discharge of the SCB. Simultaneously, an oscilloscope (44Xs, Teledyne LeCroy Inc., Los Angeles, CA, USA) recorded the current and voltage waveforms across the SCB.

### 2.3. Evaluating the Output Capacity of SCB by Principal Component Analysis

By calculating the covariance matrix of the data matrix, we obtain the eigenvalues and eigenvectors of this matrix. We then select the matrix composed of the eigenvectors corresponding to the *k* largest eigenvalues (which represent the largest variances). This transformation projects the data matrix into a new space, achieving dimensionality reduction while preserving the most significant features.

The specific process is as follows [29,30]: Let *x*_1_, *x*_2_, …, *x_p_* denote *p* feature quantities in the SCB burst process, and *c*_1_, *c*_2_, …, *c_p_* denote the weights assigned to each feature quantity. The weighted sum is defined as *s* = *c*_1_*x*_1_ + *c*_2_*x*_2_ + … + *c_p_x_p_*. The goal is to choose appropriate weights that can effectively differentiate the influence of each feature on SCB. Each voltage corresponds to a composite score denoted as *s*_1_, *s*_2_, …, *s_n_*, where *n* is the number of voltages. If these scores are widely dispersed, it indicates good differentiation. Therefore, we aim to find weights that maximize the dispersion of *s*_1_, *s*_2_, …, *s_n_*. Statistically, let *X*_1_, *X*_2_, …, *X_p_* be random variables representing sample observations. We seek *c*_1_, *c*_2_, …, *c_p_* such that the variance of *c*_1_*X*_1_ + *c*_2_*X*_2_ + … + *c_p_X_p_* is maximized. Since variance measures the degree of variation in the data, maximizing it ensures that we capture the largest variability among the p variables. To avoid trivial solutions, we impose the constraint that the sum of squared weights equals one. Under this constraint, the optimal solution is a unit vector in p-dimensional space, representing a “direction” known as the principal component direction. One principal component may not suffice to represent the original p variables; thus, we also seek the second, third, and fourth principal components, ensuring that subsequent components do not contain information already captured by previous ones.

## 3. Result and Discussion

### 3.1. Analysis of Characteristic Parameters of the SCB Outburst Process

**Figure 3:**
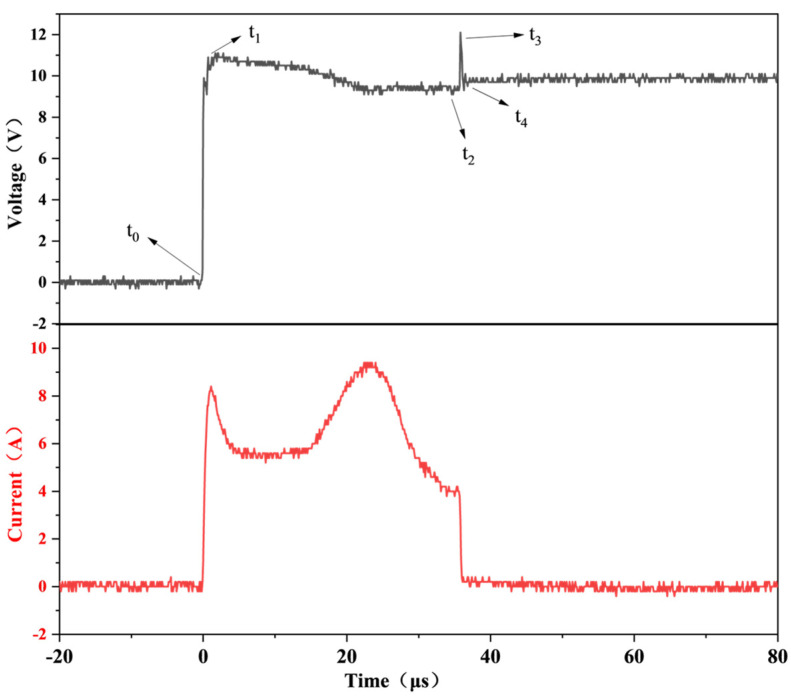
Typical electrical explosion curves and their characteristic points in SCB.

As illustrated in Figure 3, among the five key time points, *t*_0_ marks the initiation of SCB energization, *t*_1_ signifies the onset of material melting in the bridge region, *t*_2_ denotes the start of SCB vaporization, *t*_3_ represents the moment when SCB generates plasma and begins to explode. If the voltage is insufficient at this stage, plasma formation does not occur, leading to loop disconnection due to bridge vaporization, causing the current to drop to zero. *t*_4_ indicates the completion of the SCB action, with the current returning to zero. The period from *t*_0_ to *t*_3_ is termed the critical burst time (*t_d_*), during which the energy consumed by SCB is referred to as the critical burst energy (*E_d_*). The interval from *t*_0_ to *t*_4_ is defined as the total burst time (*t_s_*), and the energy expended by SCB during this phase is designated as the total burst energy (*E_s_*). The formulas for calculating *E_d_* and *E_s_* are presented below.

(1) $$E_{d}=\int_{t_{0}}^{t_{3}}UIdt$$

(2) $$E_{s}=\int_{t_{0}}^{t_{4}}UIdt$$

**Figure 4:**
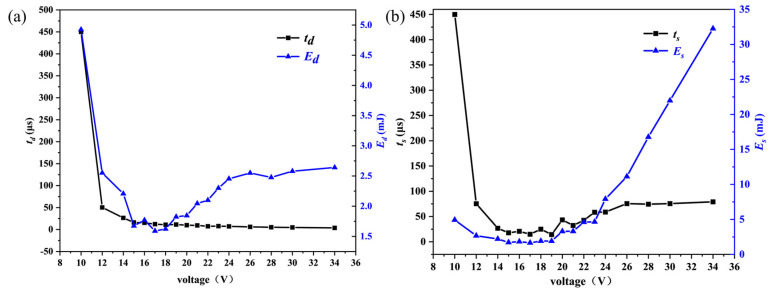
The variation of characteristic parameters of SCB with the increase of voltage during electrical explosion: (**a**) critical burst time and critical burst energy; (**b**) total burst time and total burst energy.

Figure 4 shows the characteristic parameters during the SCB explosion process. At an excitation voltage of 10 V, the bridge area temperature does not reach the melting point, resulting in a prolonged heating period. The critical burst time initially decreases with the increase in voltage. Once the voltage is sufficient to cause the SCB to fully burst, the critical burst time exhibits a slight increase and subsequently remains constant. As the voltage continues to increase, the total burst time initially decreases and subsequently increases. This phenomenon can be attributed to the fact that before reaching the critical voltage, the increased voltage is insufficient to generate plasma in the bridge area but accelerates the melting or vaporization of the bridge material, leading to circuit interruption. Consequently, the total burst time decreases. However, beyond the critical voltage, the extended duration of plasma formation causes the total explosion time to increase.

With the increase in voltage, the energy required for explosion initially decreases and then increases slightly. At 10 V, the bridge area remains in a channel state without melting, causing most of the capacitor’s stored energy to act on the SCB, thereby requiring a relatively large amount of energy for explosion. Before reaching the critical voltage, increased voltage accelerates the fusion or vaporization of the bridge area, leading to circuit disconnection and preventing the capacitor’s energy from being fully utilized by the SCB, thus reducing the energy required for explosion. After surpassing the critical voltage, the energy required for explosion increases slightly; once the voltage is sufficient for complete electrical explosion, the required energy stabilizes. Similarly, with increasing voltage, the total burst energy first decreases and then increases. At 10 V, the unmelted bridge area ensures that most of the capacitor’s energy is applied to the SCB. Before the critical voltage, higher voltages cause circuit disconnection, decreasing the total burst energy. After the critical voltage, plasma generated by the SCB’s electrical explosion conducts electricity, closing the circuit and allowing the capacitor’s energy to be released to the SCB again, resulting in an increase in total burst energy with voltage.

### 3.2. Evaluation of SCB Output Capacity by PCA

#### 3.2.1. Principal Component Extraction

To more accurately assess the output capacity of SCB, additional characteristic parameters from the SCB explosion process were identified. Specifically, three new parameters—maximum voltage, maximum current, and electric explosion energy—were incorporated in Table 1. Electric explosion energy refers to the energy involved in the gasification and plasma generation processes of SCB, and its calculation formula is as follows:

(3) $$E_{P}=E_{s}-E_{d}$$

Since at 10 V voltage, the SCB only converts electrothermal energy without involving the electrical explosion process, the data under 10 V have been excluded from the analysis. Principal component analysis was conducted on seven characteristic quantities: peak current, peak voltage, critical burst time, critical burst energy, total burst time, total burst energy, and electric burst energy. The data are normalized using z-score normalization [30]. Specifically, the mean and standard deviation of the variable are calculated. Each observation of the variable is then standardized by subtracting the mean and dividing by the standard deviation; the calculation formula is as follows:

(4) $$X_{s}=\frac{x-\bar{x}}{\sigma }$$

**Figure 5:**
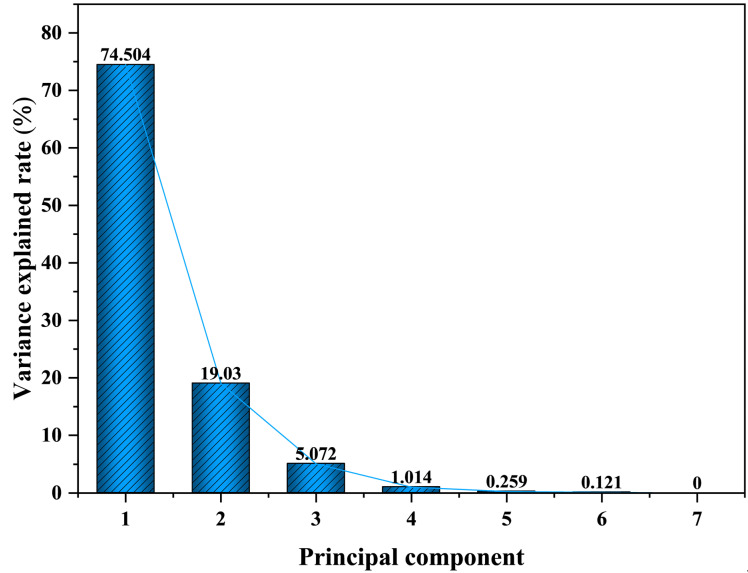
Lithotripsy: Explained variance ratio for each principal component.

The principal components of the SCB feature parameters were computed to extract the eigenvalues and variance contribution rates, as presented in Figure 5.

Table 2 presents the extracted eigenvalues of principal components and their corresponding variance explanation rates. To visually assess the variance explanation rates of the principal components, a scree plot is constructed for the principal components. As can be seen from Figure 5, Principal Component 1 (PC1) accounts for 74.5% of the original information, while Principal Component 2 (PC2) accounts for 19.03%. PC1 and PC2 can already explain about 93.5% of the original information, which indicates that PC1 and PC2 collectively retain the majority of the original information, so PC1 and PC2 are chosen to replace the original information.

#### 3.2.2. Correlation Analysis

The load coefficient signifies the correlation coefficient between PC1 and PC2 with the original standardized data. A load factor exceeding 0.5 indicates a strong association, where positive values denote a positive correlation and negative values indicate a negative correlation. As shown in Table 3, PC1 exhibits a strong positive correlation with critical electrical burst energy, total explosion time, total explosion energy, electric burst energy, critical explosion voltage, and peak current, while it shows a strong negative correlation with explosion delay time. Meanwhile, PC2 demonstrates a strong positive correlation with explosion delay time and critical electrical burst energy, a slight positive correlation with total explosion time, and slight negative correlations with other factors.

#### 3.2.3. Principal Component Composite Score [24,29]

To determine the comprehensive score for each voltage level, we begin by calculating the individual component scores.

(5) $$S_{c}=C_{lc}\times S_{d}$$

In the Formula (5), *S_c_* represents the score of the principal component, *C_lc_* represents the linear combination coefficient, and *S_d_* represents standardized data.

The linear combination coefficient (*C_lc_*) is obtained by dividing the loading coefficient by the square root of the corresponding eigenvalue.

(6) $$C_{lc}=l_{c}/\sqrt{r}$$

where *l_c_* represents the load coefficient and *r* represents the characteristic root of the corresponding principal component.

The following principal component score calculation formula is obtained:

(7) $$S_{c1}=-0.222t_{d}+0.350E_{d}+0.364t_{s}+0.415E_{s}+0.412E_{p}+0.414V_{m}+0.426C_{m}$$

(8) $$S_{c2}=0.725t_{d}+0.471E_{d}+0.420t_{s}-0.006E_{s}-0.027E_{p}-0.261V_{m}-0.482C_{m}$$

where *S_c_*_1_ and *S_c_*_2_ denote the scores of PC1 and PC2, respectively, while *V_m_* and *C_m_* represent the peak voltage and current.

**Figure 6:**
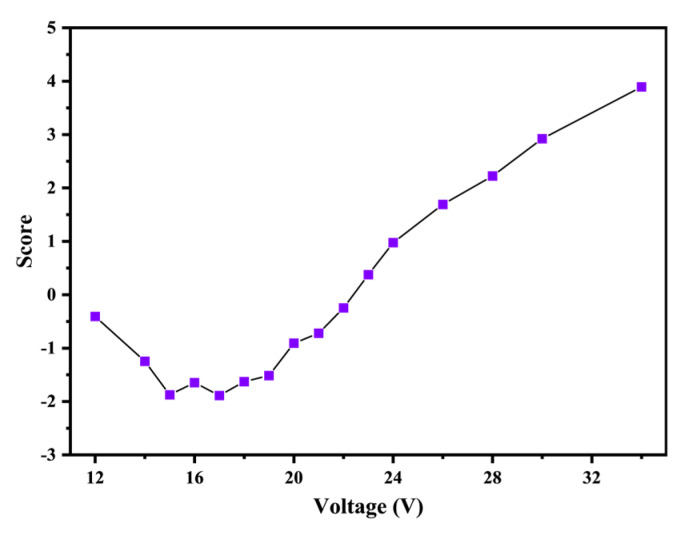
The comprehensive score of SCB output capacity under different voltages.

After obtaining the principal component scores, the comprehensive score is calculated by multiplying each principal component score by its corresponding variance contribution rate. The weighted sum of these products is then divided by the cumulative variance contribution rate.

(9) $$S=\left(74.504S_{c1}+19.030S_{c2}\right)/93.534$$

where *S* represents the comprehensive score, a higher comprehensive score indicates superior output capacity of SCB. As illustrated in Figure 6, the comprehensive score exhibits a trend of initially decreasing and subsequently increasing as the voltage rises from 10 V to 34 V, with minor fluctuations observed in the critical voltage region. The score reaches its lowest point at 17 V, indicating that this voltage corresponds to the weakest output capacity of the SCB.

### 3.3. Verification of PCA Evaluation Method

To verify the accuracy of the PCA method in evaluating the output capacity of SCB, the test system as shown in Figure 2 was utilized to trigger LTNR under various conditions, including different voltages, capacitors, and line resistances. The characteristic burst parameters of SCB were collected under these conditions. Due to the presence of the agent, only critical burst time, critical burst energy, maximum voltage, and maximum current could be measured (Table 4). The four characteristic parameters were derived through PCA, and the composite scores under various testing conditions were subsequently obtained.

To align with the characteristic parameters of the SCB that excited LTNR, three analytical factors (total burst time, total burst energy, and electric explosion energy) were excluded from Section 3.2. Consequently, a revised comprehensive score calculation model for SCB (non-igniting LTNR) was established. The SCB characteristic parameters of ignition LTNR were incorporated into the model for computation.

**Figure 7:**
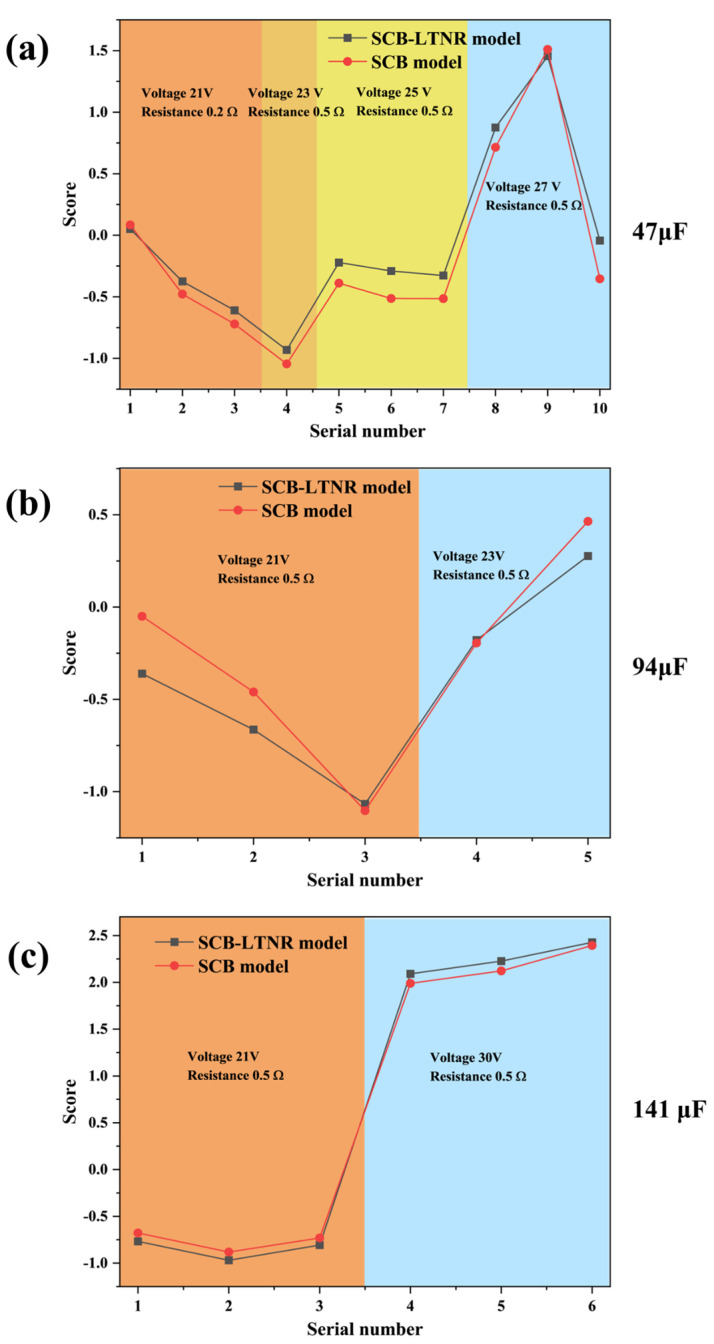
Comparison of the scores of the two models under different circuit conditions: (**a**) 47 μF; (**b**) 94 μF; (**c**) 141 μF.

By comparing the two data plots, it is evident from Figure 7 that both exhibit a similar change trend with minimal discrepancy between them. By modifying the circuit conditions, it is evident that the PCA model of SCB alone aligns well with the PCA model of SCB igniting LTNR, irrespective of alterations in capacitance, resistance, or voltage. This suggests that the PCA model demonstrates higher accuracy in predicting the SCB output ability score.

## 4. Conclusions

In this paper, a 100 µF tantalum capacitor is employed to stimulate the SCB under a steep voltage gradient. The changes in the SCB’s characteristic parameters are recorded using a digital oscilloscope, and the trends of critical burst time, critical burst energy, total burst time, and total burst energy are summarized and analyzed. Principal component analysis (PCA) is utilized to comprehensively evaluate the explosion characteristics of the SCB, thereby assessing its output capacity. The PCA model of the SCB is compared with that of LTNR triggered by the SCB, verifying the accuracy and reliability of the SCB output capacity evaluation through PCA. The main conclusions derived from this study are as follows:

The critical burst time decreases as voltage increases and remains relatively constant once the voltage reaches the level required for a complete electrical burst. Prior to reaching the critical voltage, the critical burst energy decreases with increasing voltage; however, after surpassing the critical voltage, it exhibits a slight increase with further voltage increments. Once the SCB achieves complete detonation, the critical burst energy remains largely unchanged.

The total burst time and total burst energy both decrease with increasing voltage before the critical voltage is reached, but they increase with voltage increments after the critical voltage is surpassed.

The output capacity of SCB was assessed by synthesizing the characteristic parameters of the SCB explosion process using principal component analysis. The output capacity initially decreased and subsequently increased as the voltage was raised.

The PCA model of SCB is compared with that of LTNR-fired SCB under various circuit conditions. The close agreement between the two models validates the accuracy and reliability of using PCA to assess the output capacity of SCB. PCA can effectively leverage most of the information from the SCB outbreak process to evaluate the output capacity of SCB. However, as a statistical method, PCA benefits greatly from larger datasets for more robust analysis. The limitation of this work is the insufficient number of experiments, although collecting thousands of experimental data points would require collaboration among multiple working groups. The accuracy of the PCA method should be further validated using additional energetic materials.

## Figures and Tables

**Table 1:** The change of maximum voltage, maximum current and electric explosion energy.

Voltage (V)	Maximum Voltage (V)	Electric Explosion Energy (mJ)	Maximum Current (A)
10	8.9	0	6.9
12	12.0	0.12	8.7
14	15.2	0.003	10.6
15	15.7	0.035	10.9
16	17.1	0.053	11.6
17	17.5	0.06	11.7
18	18.2	0.29	12.6
19	19.5	0.08	13.5
20	19.9	1.45	14.5
21	21.1	1.26	18.9
22	22.7	2.51	22.7
23	23.3	2.35	29.5
24	24.8	5.45	35.2
26	25.6	8.58	39.1
28	28.0	14.30	42.6
30	31.8	19.40	43.4
34	32.6	29.61	45.6

**Table 2:** Characteristic roots of principal components and variance explanation rate.

Principal Component	Principal Component Extraction		
	Characteristic Root	Variance Explanation Rate/%	Cumulative Interpretation Rate/%
1	5.215	74.504	74.504
2	1.332	19.030	93.534
3	0.355	5.072	98.606
4	0.071	1.014	99.620
5	0.018	0.259	99.879
6	0.008	0.121	100.000
7	0.000	0.000	100.000

**Table 3:** The load coefficient of the principal component.

Characteristic Parameter	PC1		PC2	
	Characteristic Root	Load Coefficient	Characteristic Root	Load Coefficient
Critical burst time	5.215	−0.508	1.332	0.837
Critical burst energy		0.8		0.544
Total burst time		0.832		0.484
Total burst energy		0.947		−0.007
Electric explosion energy		0.941		−0.031
maximum voltage		0.947		−0.301
maximum current		0.973		−0.095

**Table 4:** Electrical explosion parameters of SCB under different circuit conditions.

Capacitance (μF)	Resistance (Ω)	Voltage (V)	Critical Burst Time (μs)	Maximum Voltage (V)	Maximum Current (A)	Critical Burst Energy (mJ)
47	0.2	21	14.0	37.7	11.66	1.94
			13.3	34.6	11.66	1.81
			12.5	42.4	8	1.69
	0.5	23	13.9	35.6	8.3	1.71
		25	12	33.6	12.6	1.82
			11.6	31.2	13.3	1.8
			12.2	29.5	12.7	1.85
		27	10.2	37.2	18.6	1.93
			10.7	52.1	16.6	1.98
			9.5	35	14.3	1.74
94	0.5	21	19.3	32.1	8.67	2.12
			17.3	43.4	6.0	1.85
			15.2	40.2	7.34	1.65
		23	14.1	37.4	12	1.85
			14.7	48	9.3	1.95
141	0.5	21	16.7	37.9	8.33	1.81
			17.0	35.2	7.0	1.83
			16.2	39.4	7.3	1.79
		30	7.7	53.5	22.3	1.93
			7.5	54.9	23.3	1.92
			8.2	44.3	23.0	2.2

## Data Availability

Data available on request from the authors.

## References

[1] Zvulun E., Toker G., Gurovich V.T., Krasik Y.E. (2014). Shockwave generation by a semiconductor bridge operation in water. J. Appl. Phys..

[2] Benson D.A., Larsen M.E., Renlund A.M., Trott W.M., Bickes R.W. (1987). Semiconductor bridge: A plasma generator for the ignition of explosives. J. Appl. Phys..

[3] Rossi C., Larangot B., Lagrange D., Chaalane A. (2005). Final characterizations of MEMS based pyrotechnical microthrusters. Sens. Actuators A Phys..

[4] Hollander L.E. (1968). Semiconductive Explosive Igniter. U.S. Patent.

[5] Wang J., Zhou B., Ye S., Chen H. (2020). Novel electro-explosive device incorporating a planar transient suppression diode. IEEE Electron Device Lett..

[6] Li H., Zhou Q., Ren H., Jiao Q., Du S., Yang G. (2016). Ignition characteristics of semiconductor bridge based on lead styphnate and lead azide charges under capacitor discharge conditions. Sens. Actuators A Phys..

[7] Yi Z., Cao Y., Yuan J., Mary C., Wan Z., Li Y., Zhang C., Zhang L., Zhu S. (2020). Functionalized carbon fibers assembly with Al/Bi_2_O_3_: A new strategy for high-reliability ignition. Chem. Eng. J..

[8] Zuo B., Zheng X., Feng C., Yan S., Li F., Peng J., He C., Huang L., Chen L. (2023). Investigation on heat transfer mechanism of semiconductor bridge ignition based on thermoelectric coupling analysis. J. Mater. Today Commun..

[9] Chen J., Zhang X. (2019). Investigations of electrical and thermal properties in semiconductor device based on a thermoelectrical model. J. Mater. Sci..

[10] Turcotte R., Goldthorp S., Badeen C.M., Chan S.K. (2008). Hot-wire ignition of AN-based emulsions. Propellants Explos. Pyrotech..

[11] Kim J.U., Park C.O., Park M., Kim S.H., Lee J.B. (2002). Characteristics of semiconductor bridge (SCB) plasma generated in a micro-electro-mechanical system (MEMS). Phys. Lett. A.

[12] Lee K.N., Park M.I., Choi S.H., Park C.O., Uhm H.S., Kye-Nam L., Myung-Il P., SungHo C., Chong-Ook P., Uhm H.S. (2002). Characteristics of plasma generated by polysilicon semiconductor bridge (SCB). Sens. Actuators A Phys..

[13] Xu J., Zhou Y., Shen Y., Wang Y., Ye Y., Shen R. (2024). Identifying the enhancement mechanism of Al/MoO_3_ reactive multilayered films on the ignition ability of semiconductor bridge using a one-dimensional gas-solid two-phase flow model. J. Defence Technol..

[14] Xu J., Zhou Y., Shen Y., Wang C., Wang Y., Ye Y., Shen R. (2022). Characteristics of micro energetic semiconductor bridge initiator by depositing Al/MoO_3_ reactive multilayered films on micro bridge with different bridge size. J. Sens. Actuators A Phys..

[15] Xu J., Tai Y., Shen Y., Xu W., Ye Y., Shen R., Hu Y. (2019). Characteristics of energetic semiconductor bridge initiator based on different stoichiometric ratios of Al/MoO_3_ reactive multilayer films under capacitor discharge conditions. J. Sens. Actuators A Phys..

[16] Wang R., Zhu S., Sun Q., Xu Z. (2021). Research of the Characteristics of Semiconductor Bridge (SCB) Plasma. J. Fusion Sci. Technol..

[17] Xu J., Tan J., Li H., Ye Y., Chen D. (2021). Modeling the Heating Dynamics of a Semiconductor Bridge Initiator with Deep Neural Network. J. Micromach..

[18] Sirakov N.M., Shahnewaz T., Nakhmani A. (2024). Training Data Augmentation with Data Distilled by Principal Component Analysis. J. Electron..

[19] Joshua W.B., James B., Jen H., Lynn C. (2022). Using principal component analysis to explore co-variation of vowels. J. Lang. Linguist. Compass.

[20] Zhang Y., Xian H., Rao C. (2023). Dispersed fringe cophasing method based on principal component analysis. J. Opt. Lett..

[21] Zhang S., Gong L., Gao W., Zeng Q., Xiao F., Liu Z., Lei J. (2023). weighted mean temperature model using principal component analysis for Greenland. J. GPS Solut..

[22] Hotelling H. (1933). Analysis of a complex of statistical variables into principal components. J. Educ. Psychol..

[23] Greenacre M., Groenen P.J.F., Hastie T., D’enza A.I., Markos A., Tuzhilina E. (2022). Principal component analysis. J. Nat. Rev. Methods Primers.

[24] Bro R., Smilde A.K. (2014). Principal component analysis. J. Anal. Methods.

[25] Nakao E.K., Levada A.L. (2024). Information theory divergences in principal component analysis. J. Pattern Anal. Appl..

[26] Kang Z., Liu H., Li J., Zhu X., Tian L. (2023). Self-paced principal component analysis. J. Pattern Recognit..

[27] Fraino P.E. (2023). Using principal component analysis to explore multi-variable relationships. J. Nat. Rev. Earth Environ..

[28] Tonin F., Tao Q., Patrinos P., Suykens J. (2024). Deep Kernel Principal Component Analysis for multi-level feature learning. J. Neural Netw..

[29] Mackiewicz A., Ratajczak W. (1993). Principal Components Analysis (PCA). J. Comput. Geosci..

[30] Al Shalabi L., Shaaban Z., Kasasbeh B. (2006). Data mining: A preprocessing engine. J. Comput. Sci..

